# Circumcision

**Published:** 1899-04

**Authors:** Thomas H. Hancock

**Affiliations:** Atlanta, Ga.


### CIRCUMCISION.*

By THOS. H. HANCOCK, M.D.,

ATLANTA, GA.

Circumcision was instituted by God in his covenant with Abraham, 1910 B. C., when he commanded him and every man child in his house to be circumcised, and after that to circumcise every male child when he was eight days old, and it was done. This is the first record we have of an operation being performed by man. The record is very incomplete, as it does not mention what kind of instrument was employed, where the incision was made, or whether ligatures, sutures, anesthetics or antiseptics were used or not. Abraham was ninety-nine years old when he parted with his foreskin. In the year 1451 B. C., the children of Israel having neglected this rite for the preceding forty years, during their sojourn in the wilderness, God commanded Joshua to make him sharp knives and to circumcise all of the uncircumcised at the hilt of their foreskins.

*Read before the Atlanta Society of Medicine.

The man who performs this ceremony among the Hebrews is called a Mohl. It seems to be the general impression among the Gentiles that the Rabbi performs it, and we hear all kinds of tales about the way he stops the hemorrhage. Any Hebrew who knows how to perform this operation can be a Mohl. There is nothing in the duties of a Rabbi which requires him to do it, and, as a matter of fact, very few of them can do it. I attended a Hebrew circumcision some time ago, and it was performed in the following manner: At the appointed time the Mohl and his assistant came in, the invited guests (no females being allowed on such occasions) were already present, all the Hebrews wearing their hats, and all the Gentiles bareheaded. The baby, a very robust one, was given six drops of paregoric and in a few minutes the father brought him in on a pillow, which was placed on a table before the Mohl, who wore a red sash around his neck, crossed over his breast, passed beneath his arms, and tied at the back. The Mohl removed the napkin (all the while speaking Hebrew), pulled the foreskin into the notch of the shield (a flat piece of silver shaped something like the handle of a grooved director, but much larger) and in a few seconds (amid a voluminous flow of Hebrew) the foreskin was cut off with a sharp scalpel close down to the shield, and a pair of sharp thumb-nails quickly assisted in breaking up the adhesions and pulling the mucous membrane back from the glans. A sugar-teat was placed in the mouth of the youngster to stop his crying. Some sour wine and vinegar had been mixed in a silver cup and this was squirted from the mouth of the Mohl on the wound, very much like a Chinaman squirts water on a shirt-bosom while he is ironing it. Then numerous pledgets of soft lint, soaked in the same mixture, were wound about the penis until a large roll was made. The napkin was replaced, the Mohl washed his hands and the guests repaired to the dining-hall where a feast was held, toasts drank to the health of the child, and the ceremony was finished.

It seems to have been a pretty serious operation for adults before the days of antiseptics, for when Shechem ravished Dinah, Jacob persuaded the Shechemites to be circumcised, and on the third day thereafter Simeon and Levi (her brothers) were able to kill every one of them. This operation should be done when the child is about eight days old, when it is a very simple thing, but if it is deferred until youth or manhood it is more trouble, the vessels requiring to be ligated, the wound to be sutured, and some local anesthetic to be given. I think a general anesthetic is unjustifiable except for children.

I have found the following method for this operation on adults to be very satisfactory: The glans and the entire penis is washed thoroughly with soap and water, then a bichloride or carbolized solution. A tape is then tied around the organ near its base tight enough to retard the venous return of blood, and about half a drachm of a two to a four per cent. solution of cocaine or eucaine is injected by inserting a hypodermic needle through the mucous membrane on the upper part of the foreskin near the preputial opening and injecting to either side as it is carried back between the mucous membrane and the integument to a point a little behind the proposed line of incision. The needle is then carried around the side which is to be removed (for I remove only one-half of the foreskin at one operation) injecting the solution along the line of amputation until the frenum is reached. A sharp-pointed curved bistoury is now passed between the upper surface of the glans and the prepuce in the median line (the cutting edge being upwards) until the point is at the line of amputation, when it is passed through the integument, and the prepuce is split from behind forward. The side which we propose to remove is caught with a clamp near the line of this incision, then the mucous membrane on the other side of the incision is sewed to the integument on that side with a continuous catgut suture. The side which has been anesthetized, and which is clamped, is then trimmed off with a pair of scissors, all bleeding vessels ligated, and the mucous membrane sewed to the integument along the line of incision with a continuous catgut suture. The tape is then removed, the wound washed in an antiseptic solution, and a plain sterilized gauze dressing applied. When the wound has completely healed, the other side should be cleansed, anesthetised, and removed in the same way. By removing only one-half of the prepuce at a time, the circulation is not so seriously interfered with, and there is usually very little swelling, ecchymosis, or priapism. The patient needs only to be quiet for a few hours after the operation. He can then go about his business without pain or soreness, and when night comes he can sleep.

Now, concerning the advantages derived from circumcision. If it is done in early babyhood, phimosis and paraphimosis will be done away with. There are numerous diseases which are claimed to be caused by the irritation produced by tight prepuces through reflex action. Cleanliness has nothing to do with godliness, but it is a good thing, and circumcision certainly helps in this direction. It makes one less liable to contract syphilis or chancroid, and the last and chief reason why it should be performed is because it breaks or weakens the nerve connection between a man's mind and this organ.

There may be some here who will take issue with me on these points and who will say that circumcision offers no advantages, but not one of these will talk from experience.

